# RV144-like trial in macaques using ALVAC-SIV & gp120 induces innate immunity and increases the frequency of NK22 & NKG2A+ cells in mucosal tissues

**DOI:** 10.1186/1742-4690-9-S2-O14

**Published:** 2012-09-13

**Authors:** NP Liyanage, P Pegu, S Gordon, M Cameron, K Foulds, M Doster, M Vaccari, R Koup, M Roederer, R Sékaly, G Franchini

**Affiliations:** 1 Animal Models & Retroviral Vaccines Section , Bethesda, MD, USA; 2 Vaccine & Gene Therapy Institute -Florida, FL, USA; 3 Vaccine Research Center, NIAID, NIH, Bethesda, MD, USA

## Background

NK cells play a pivotal role in the innate immunity and patrol various tissues, including mucosal sites, the portal of entry of HIV. We recently reproduced in the SIV _mac251-_ rhesus macaque (RM) model the limited protection reported in the RV144 HIV vaccine trial in humans (32% protection from HIV acquisition), using similar vaccines (ALVAC-SIV & gp120).

## Methods

In here, we immunized, by the intramuscular route, 6 RM with 10^8^ PFU of ALVAC-SIV at weeks, 0 (V0), 4 (V2), and with both ALVAC SIV and the SIVgp120 envelope proteins, adjuvanted with ALUM at week 12 (V3) and 24 (V4) and analyzed the profile of gene expression at 24 hours after each vaccination. In parallel, we studied the phenotypical and functional changes in the NK cell subsets after vaccination by multi parametric flow cytometry.

## Results

Microarray analysis in the PBMC after V1 and V2 showed the up regulation of anti viral (MX1, HERC-5) and IFN responsive genes and a down regulation of pro-inflammatory genes. Significant alteration in the gene expression profile was also observed after the gp120/Alum boost (V3). Interestingly, NK cells associated genes were up regulated after V3 vaccination. These finding paralleled our results observed by FACS analysis that demonstrated an increased frequency of NK22 cells at mucosal sites. These NK22 cells expressed CCR6 (a gut homing marker) and are thought to play a role in mucosal immunity. Similarly, vaccination also increased the frequency of NKG2A+ cells that were either cytotoxic (CD107a+) or cytokine producing (IFNg+).

## Conclusion

Thus, the ALVAC SIV/gp120 vaccine regimen induces a significant activation of NK cells and other innate immune responses. Given that this vaccine platform has conferred some degree of protection from HIV infection in humans, understanding the role of innate immunity in protection from SIVmac251 may guide our effort in the development of novel vaccine strategies against HIV.

